# How to Prevent Ventilator-Induced Lung Injury in Intraoperative Mechanical Ventilation? A Randomized Prospective Study

**DOI:** 10.4274/TJAR.2024.241426

**Published:** 2024-07-12

**Authors:** Mesut Türk, Furkan Tontu, Sinan Aşar, Nalan Saygı Emir, Gülsüm Oya Hergünsel

**Affiliations:** 1 Ağrı Training and Research Hospital, Clinic of Anaesthesiology and Reanimation, Ağrı, Turkey; 2 University of Health Sciences Turkey, Başakşehir Çam and Sakura City Hospital, Clinic of Anaesthesiology and Reanimation, İstanbul, Turkey; 3 University of Health Sciences Turkey, Bakırköy Dr. Sadi Konuk Training and Research Hospital, Clinic of Anaesthesiology and Reanimation, İstanbul, Turkey

**Keywords:** Lung injury, mechanical power, mechanical ventilator, perioperative care, prone position, supine position

## Abstract

**Objective:**

Intraoperative mechanical ventilation practices can lead to ventilator-induced lung injury (VILI) and postoperative pulmonary complications in healthy lungs. Mechanical power (MP) has been developed as a new concept in reducing the risk of postoperative pulmonary complications as it considers all respiratory mechanics that cause VILI. The most commonly used intraoperative modes are volume control ventilation (VCV) and pressure control ventilation (PCV). In this study, VCV and PCV modes were compared in terms of respiratory mechanics in patients operated in the supine and prone positions.

**Methods:**

The patients were divided into 4 groups (80 patients), volume control supine and prone, pressure control supine and prone with 20 patients each. MP, respiratory rate, positive end-expiratory pressure, tidal volume, peak pressure, plato pressure, driving pressure, inspiratory time, height, age, gender, body mass index, and predictive body weight data of the patients included in the groups have been obtained from “electronic data pool” with Structured Query Language queries.

**Results:**

The supine and prone MP values of the VCV group were statistically significantly lower than the PCV group (*P* values were 0.010 and 0.001, respectively).

**Conclusion:**

Supine and prone MP values of the VCV group were calculated significantly lower than the PCV group. Intraoperative PCV may be considered disadvantageous regarding the risk of VILI in the supine and prone positions.

**Main Points**

⦁ Intraoperative mechanical ventilation can cause ventilator-induced lung injury (VILI).

⦁ Increased mechanical power may be associated with postoperative pulmonary complications.

⦁ Volume control mode may have some advantages over pressure control mode in reducing intraoperative VILI.

## Introduction

Although mechanical ventilation is a life-saving intervention, it can lead to ventilator-induced lung injury (VILI). VILI is the damage caused by positive pressure ventilation that starts with the use of mechanical ventilators.^1^ Many factors cause VILI such as tidal volume, driving pressure, flow, respiratory rate, and positive end-expiratory pressure (PEEP). Mechanical power (MP), which collects these different variables in a single parameter, offers us new possibilities in predicting VILI at the bedside.^2, 3, 4, 5, 6^ The MP is above a certain threshold causes damage ranging from pulmonary parenchymal rupture to severe inflammation and edema.^5, 7^ Also, higher MP values are associated with higher mortality.^8^ The protective ventilation strategy in intensive care units is also applied in operating rooms (ORs) to minimize the risk of postoperative pulmonary complications due to VILI.^9^ While the volume control mode was at the forefront of the old anaesthesia devices used in the OR, today there are anaesthesia devices with many modes and features, as in intensive care units. This confuses the use of mechanical ventilators in the perioperative period. Although the respiratory parameters (tidal volume, drive pressure and respiratory rate) that contribute to the calculation of MP are similar, lower power values are calculated in volume control ventilation (VCV) mode compared to pressure control ventilation (PCV) in acute respiratory distress syndrome (ARDS) patients.^10^ However, in a study comparing the MP values of ventilation modes (VCV and PCV) in patients undergoing laparoscopic surgery, lower MP values were observed in the PCV mode.^11^ The hypothesis of our study posits that lower MP values will be observed in the VCV mode in both prone and supine positions for patients with healthy lungs undergoing elective surgery. The primary outcome variable of this study is the MP values of PCV and VCV modes in both prone and supine positions.

## Methods

### Study Design and Population

This prospective randomized controlled observational clinical study was approved by the University of Health Sciences Turkey, Bakırköy Dr. Sadi Konuk Training and Research Hospital, Clinical Research Ethics Committee (approval no.: 2019-15-04, date: 05.08.2019). The study has been registered on ClinicalTrials.gov (decision number: NCT05814081).

Cervical hernia, lumbar hernia, and lumbar stabilization cases who were operated in the neurosurgery OR of the anaesthesia and reanimation clinic were examined.

The patients (n=80) were divided into four groups;

PCV Group

Subgroup A: PCV Supine Group (20 patients).

Subgroup B: PCV Prone Group (20 patients).

VCV Group

Subgroup A: VCV Supine Group (20 patients).

Subgroup B: VCV Prone Group (20 patients).

All patients were seen one day before the operation, their anamnesis was taken, physical examinations were made, and vital signs and laboratory measurements were evaluated. Informed consent was obtained from all patients before the operation.

Electrocardiogram, non-invasive blood pressure measurement, and SpO_2_ monitoring were provided to the patients who were taken to the OR. Vascular access was established with a 20 G intravenous cannula. An infusion of 4-5 mL kg^-1^ h^-1^ balanced fluid (isolyte, ringer lactate) was started. Balanced anaesthesia was applied to all patients. Ventilation of all patients was adjusted to be 45% O_2_/air, flow 3 Lt/min, respiratory rate 12 breaths/min, PEEP: 5 cmH_2_O, tidal volume 6-8 mL kg^-1^ according to predicted body weight (PBW), inspiration/expiration time ratio 1:2 and SpO_2_: 92-96% was targeted. Ventilation was provided in PCV mode with a tidal volume of 6-8 mL kg^-1^ per kilogram of PBW while maintaining constant driving pressure values with the VCV group. All patients were ventilated with the Maquet Flow-i (Sweden) anaesthesia device. Patient data were recorded on the anaesthesia device ventilator at a sweep speed of 20 mm s. At the end of the operation, when spontaneous breathing started, 0.01 mg kg^-1^ atropine and 0.03 mg kg^-1^ neostigmine were administered. The patients were extubated when spontaneous respiration was sufficient. Patients taken to the postoperative recovery room were observed here, and patients with a Modified Aldrete Score above 9 were sent to the service. Other drugs administered during the operation and intraoperative complications were recorded. All respiratory parameters and other vital signs of the patients were recorded electronically at 1-minute intervals with the ImdSoft/Metavision (Canada) software system, which is used as clinical decision support software in our OR.

Respiratory system power values in VCV were calculated with the practical power formula of Gattinoni et al.,^6^ which was predefined in the software [(MPvcv(simp.) = 0.098 x ∆V x RR (Ppeak- ½ x ∆Pinsp)]. For PCV, power was calculated with the pressure control practical power equation developed by Becher et al.^12^ [(MPpcv(simp.) = 0.098 x RR x ∆V x (∆Pinsp + PEEP)].

In VCV, Pplato is calculated automatically by the ventilator with an inspiratory pause. Since such an adjustment cannot be made in PCV due to the variable gas flow, Pplato pressure cannot be obtained without hold maneuvers. However, in the pressure control mode, the Ppeak pressure is considered equal to Pplato, since the driver pressure is fixed in advance.

In PCV, if there is no chronic obstructive pulmonary disease (COPD) and bronchoalveolar fistula and PEEP is zero EEP, the peak inspiratory pressure (Ppeak) and the alveolar pressure (Pplato) were considered equal because the airway pressure (∆Pinsp) at the end of inspiration was fixed beforehand (Pplato = Ppeak).^13, 14^

Pplato = Ppeak = ∆Pinsp. In the presence of PEEP, the airway pressure (Ppeak = Pplato) will be ∆Pinsp + PEEP at the end of inspiration. DeltaP = ∆Pinsp = Pplato-PEEP = Ppeak-PEEP. Cdyn = ∆V/(Ppeak-PEEP) and Cstatic = ∆V/(Ppalto-PEEP), Cstatic = Cdyn = Compliance.

### Inclusion Criteria

• American Society of Anesthesiologists I-III risk group patients.

• Patients between the ages of 18-70.

• At least 2 hours of mechanical ventilation time.

### Exclusion Criteria

• Patients with COPD or asthma bronchial.

• Patients with a functional capacity of less than 7 Measurement of Exercise Tolerance before Surgery.

• Pregnant and lactating female patients.

• Patients who have had thoracic surgery before.

• Patients with body mass index (BMI) above 35.

• Patients who had hemodynamic instability or desaturation (SpO_2_<92%) during the operation.

• Patients with hemorrhage.

### Obtaining Patient Data

The respiratory parameters (respiratory rate, PEEP, TVe, Ppeak, Pplato, driving pressure, Tinsp, etc.) of the patients included in the study were taken from the Maquet Flow-i (Sweden) anaesthesia machine and recorded in the “ImdSoft-Metavision/QlinICU Clinical Decision Support Software” (Canada) data pool. Patients’ demographic information (height, age, gender, BMI, pedicure body weight) and minute mechanical ventilator data were obtained from the data pool using Structured Query Language queries. Statistical analyses were made after taking the mean values of every parameter in excel.

### Statistical Analysis

The homogeneity of the data was evaluated with the Shapiro-Wilk test. Student’s t-test and Mann-Whitney U test were used for pairwise comparison of data. The chi-square test was used in the comparison of qualitative data. The Kruskal-Wallis test was used for multiple group comparisons. Mean ± standard deviation (SD) values are based on statistical representation. Values with *P *< 0.05 were considered statistically significant. Statistical analyses were made with the GraphPad Prism V5.01 (San Diego, California, USA) program.

120-minute measurements were made for each patient. A total of 2,400 minutes of measurements were made for 20 patients in each group. Data were saved in ImdSoftMetavision/QlinICU Clinical Decision Support Software. Statistical analyses were performed based on patient averages. In the preliminary statistical analyses performed with 10 patients, the mean MP difference between the VCV group and the PCV group patients was calculated as 2 J/minute and the standard deviation as 2.5 J/minute. The number of patients required to be included in each group was calculated as 20 for the power of the study to be over 80% with alfa 0.5 error and 95% confidence interval (G*power version 3).

## Results

In this study, there was no significant difference between the groups’ demographic data, [including BMI, operation duration, length of stay in hospital, perioperative fluid admission, and Assess Respiratory Risk in Surgical Patients in Catalonia (ARISCAT) score]. The mean ± SD and *P *values of the groups are shown in Table 1.

A statistically significant difference was observed between the mean values of the supine MPrs, Ppeak, and TVe of the VCV and PCV groups. The *P *values were calculated as 0.010, 0.024, and 0.001, respectively. No statistically significant difference was observed between the mean values of Pplato, Cstatic, Cdyn, and Tinsp.

A statistically significant difference was observed between the mean values of prone MPrs and TVe of the VCV and PCV groups. *P *values were calculated as 0.001 and 0.011, respectively. No statistically significant difference was observed between the mean values of Cstatic, Tinsp, Pplato, Cdyn, and Ppeak.

The supine and prone mean ± SD and *P *values of the above-mentioned respiratory parameters of the VCV and PCV groups are shown in Table 2.

No statistically significant difference was observed between the mean values of MPrs, Ppeak, Pplato, Cstatic, TVe, Cdyn, and Tinsp of the VCV supine and prone groups. This is accurate for the PCV supine and prone groups.

The supine and prone mean ± SD and *P *values of the above-mentioned respiratory parameters of the VCV and PCV groups are shown in Table 3.

## Discussion

In the old anaesthesia devices used in the OR, the VCV mode was at the forefront. Today, there are anaesthesia devices with many modes and features, as in the intensive care units (ICUs). This confuses the use of mechanical ventilators. VCV, PCV, and many different modes are used in ICU. The primary outcome of the study is to calculate and compare the MP values of the PCV and VCV groups in both supine and prone positions.

In this study, the expiratory tidal volume TVe and Ppeak values of the PCV group in the supine position were found to be lower than the values of the VCV group. There was no difference between the Pplato, PEEP, DP values. Although TVe and Ppeak was low in PCV, the MP was calculated higher. The P-V loop where the power is calculated in both ventilation modes is different. The volume control P-V loop is triangular, while the pressure control P-V loop is square. This difference is due to the high inspiratory resistance created by the variable gas flow in the PCV.^15^ Again, due to this variable gas flow in PCV, inspiratory resistance values cannot be measured. Therefore, the inspiratory resistances of the two ventilation modes could not be objectively compared. However, the gas flow pattern (decelerating flow) in PCV and the high power calculations measured despite similar respiratory parameters compared to VCV, place PCV at a distinct disadvantage.^16^ In addition, in the PCV, rapid transmission of per cycle energy to the lungs in early inspiration may increase the damage.^17^ However, the most accurate formula for calculating power in pressure control mode is still controversial. The simplified MPpcv formula used in this study calculates high MP values according to the geometric method.^17^ The margin of error in MP calculated by the MPpcv(simp.) formula and the relationship between the decelerating gas flow pattern in PCV and MP need to be clarified. For this reason, the statement that the PCV mode is disadvantageous compared to the VCV mode may have been premature.

In VCV, a higher Ppeak is needed to maintain the same tidal volume set in the supine position, and also in the prone position. This is the reason for the difference in Ppeak values between the two ventilation modes in the prone position. It is also known that in the prone position, since the thoracic wall motion is limited, thoracic compliance decreases and Ppeak values increase.^18^^, ^^19^

There was no difference in respiratory mechanics values (including MP) between the supine and prone positions in both VCV and PCV modes. In a paper presented at the American Thoracic Society conference in 2018, MP was found to decrease in ARDS patients after a prone position of at least 8 hours.^20^ This condition, which is due to alveolar recruitment, is not seen in this patient group with normal lungs in the OR.

There was no difference in the postoperative pulmonary complication score (ARISCAT score) of the VCV and PCV modes in the supine and prone positions. In all groups, the MP values were calculated far below 17 J, which was determined as the threshold MP value for the risk of VILI for patients with healthy lung.^8^

Therefore, the risk of postoperative pulmonary complications is not predicted with the applied MP values.

MP as defined here relates to the inspiratory phase. All the energy accumulated in end-inspiration must be dissipated to the lung tissue and atmosphere when exhalation is complete. It is not clear whether controlling the potentially important expiratory flow will help reduce VILI. However, it is estimated to have a damaging effect in the early expiratory phase.^7^ This energy in the early expiratory phase cannot currently be calculated in any ventilator mode.

There was no significant difference between the BMI values of the patient groups.

### Study Limitations

Since VCV and PCV modes are frequently compared with arterial blood gases in the literature, blood gas measurements were not evaluated.

## Conclusion

It is challenging to ascertain the superiority of one mechanical ventilation mode over another.However, MP values are lower in VCV compared to PCV in both prone and supine positions. In addition, all respiratory mechanics in VCV mode can be obtained easily without the need for hold maneuvers (Pplato, Cstatic, etc.), and learning VCV is simpler.

## Ethics

**Ethics Committee Approval:** This prospective randomized controlled observational clinical study was approved by the University of Health Sciences Turkey, Bakırköy Dr. Sadi Konuk Training and Research Hospital, Clinical Research Ethics Committee (approval no.: 2019-15-04, date: 05.08.2019).

**Informed Consent:** Informed consent was obtained from all patients before the operation.

## Figures and Tables

**Table 1. Demographic Data of Patients:** 

	**VCV** **Prone (n=20)** **(Mean ± SD)**	**VCV** **Supine (n=20)** **(Mean ± SD)**	**PCV** **Prone (n=20)** **(Mean ± SD)**	**PCV** **Supine (n=20)** **(Mean ± SD)**	***P* value**
**Gender, female (%)**	13 (65)	13 (65)	11 (55)	7 (35)	0.1
**Weight (kg)**	75.5±9.6	71.3±10.9	75.4±10.6	74.7±10.2	0.9
**Height (cm)**	168±9	168±11	171±9	171±10	0.6
**PBW (cm)**	64.6±10.9	60.8±10.9	64.2±11.2	66.2±115	0.4
**BMI (kg/m^2^)**	26.5±4.2	25.2±2.3	25.7±.3.6	25.3±2.9	0.07
**Operation time (hours)**	2.6±0.9	2.9±1.0	3.1±1.7	2.5±0.9	0.4
**Length of stay in hospital (days)**	3.1±1.2	3.3±1.4	3.2±1.2	3.4±1.6	0.8
**Peroperative given fluid (mL)**	1640±636	1755±705	1810±1190	1510±786	0.6
**ARISCAT score**	20±5	22±6	23±8	21±8	0.6

The chi-square test was used to determine the high-risk group category and gender percentage and significance level shown in the table between the groups, and the Kruskal-Wallis test was used for the analysis of other parameters.

ARISCAT, Assess Respiratory Risk in Surgical Patients in Catalonia; BMI, body mass index; PBW, predictive body weight; PCV, pressure control ventilation; VCV, volume control ventilation.

**Table 2. Respiratory Parameters:** 

**VCV vs. PCV**	**VCV supine vs. PCV supine** **(Mean ± SD)**	***P* value**	**VCV prone vs. PCV prone** **(Mean ± SD)**	***P* value**
MPrs, J min	7.4±2.0 vs. 9.7±2.7	**0.010**	7.9±2.0 vs. 10.9±3.0	**0.001**
Pplato, cmH_2_O	15.1±2.5 vs. 16.0±3.3	0.78	17.4±4.5 vs. 17.4±3.2	0.9
Ppeak, cmH_2_O	18.1±3.8 vs. 16.0±3.3	**0.024**	20.1±4.8 vs. 17.4±3.2	0.6
TVe, mL	479±37 vs. 423±58	**0.001**	478±20 vs. 428±59	**0.011**
Cdyn, mL cmH_2_O	40.5±12 vs. 43.5±11	0.3	34.8±9.5 vs. 37.1±8.2	0.3
Cstatic, mL cmH_2_O	45.7±25.3 vs. 43.5±11	0.3	44.0±15.5 vs. 42.5±10	0.4
Tinsp, second	4.3±0.7 vs. 4.4±0.8	0.4	4.4±0.8 vs. 4.3±1.1	0.2

Statistical analysis of the respiratory parameters of the supine and prone positions of the VCV and PCV groups were performed with the Mann-Whitney U test.

Cstatic, static compliance; Cdyn, dynamic compliance; MPrs, respiratory system mechanical power; PCV, pressure   control ventilation; PEEP, positive end-expiratory pressure; Ppeak, peak   inspiratory pressure; Pplato, plateau pressure; Tinsp, inspiratory time; TVe, expiratory tidal volume; VCV, volume control ventilation.

**Table 3. Respiratory Parameters:** 

**Supine vs. prone**	**VCV supine vs VCV prone**	***P* value**	**PCV supine vs PCV prone**	***P* value**
MPrs, J min	7.4±2.0 vs. 7.9±2.0	0.5	9.7±2.7 vs. 10.9±3.0	0.1
Pplato, cmH_2_O	15.1±2.5 vs. 7.4±4.5	0.07	16.0±3.3 vs. 17.4±3.2	0.61
Ppeak, cmH_2_O	18.1±3.8 vs. 20.1±4.8	0.2	16.0±3.3 vs. 17.4±3.2	0.61
TVe, mL	479±37 vs. 478±20	0.8	423±58 vs. 428±59	0.7
Cdyn, mL cmH_2_O	40.5±12 vs. 34.8±9.5	0.1	43.5±11 vs. 37.1±8.2	0.06
Cstatic, mL cmH_2_O	45.7±25.3 vs. 44.0±15.5	0.07	43.5±11 vs. 42.5±10	0.8
Tinsp, second	4.1±0.4 vs. 4.1±0.4	0.9	4.4±0.8 vs. 4.3±1.1	0.8

Statistical analysis of the supine and prone positions’ parameters of the VCV group and the supine and prone positions’ parameters of the PCV group were performed with the Mann-Whitney U test.

Cstatic, static compliance; Cdyn, dynamic compliance; MPrs, respiratory system mechanical power; PCV, pressure control ventilation;   Ppeak, peak inspiratory pressure; Pplato, plateau pressure; TVe, expiratory tidal volume; VCV, volume control ventilation.
